# Characterization of an active LINE-1 in the naked mole-rat genome

**DOI:** 10.1038/s41598-021-84962-8

**Published:** 2021-03-11

**Authors:** Shunichi Yamaguchi, Shizuka Nohara, Yuki Nishikawa, Yusuke Suzuki, Yoshimi Kawamura, Kyoko Miura, Keizo Tomonaga, Keiji Ueda, Tomoyuki Honda

**Affiliations:** 1 Division of Virology, Department of Microbiology and Immunology, Osaka University Graduate School of Medicine, 2-2 Yamada-oka, Suita, Osaka 565-0871 Japan; 2 Laboratory of RNA Viruses, Department of Virus Research, Institute for Frontier Life and Medical Sciences (InFRONT), Kyoto University, Kyoto, Japan; 3 Department of Aging and Longevity Research, Kumamoto University, Kumamoto, Japan; 4 Center for Metabolic Regulation of Healthy Aging, Kumamoto University, Kumamoto, Japan; 5 Laboratory of RNA Viruses, Graduate School of Biostudies, Kyoto University, Kyoto, Japan; 6 Department of Molecular Virology, Graduate School of Medicine, Kyoto University, Kyoto, Japan

**Keywords:** Gene expression, Transposition

## Abstract

Naked mole-rats (NMRs, *Heterocephalus glaber*) are the longest-living rodent species. A reason for their long lifespan is pronounced cancer resistance. Therefore, researchers believe that NMRs have unknown secrets of cancer resistance and seek to find them. Here, to reveal the secrets, we noticed a retrotransposon, long interspersed nuclear element 1 (L1). L1s can amplify themselves and are considered endogenous oncogenic mutagens. Since the NMR genome contains fewer L1-derived sequences than other mammalian genomes, we reasoned that the retrotransposition activity of L1s in the NMR genome is lower than those in other mammalian genomes. In this study, we successfully cloned an intact L1 from the NMR genome and named it NMR-L1. An L1 retrotransposition assay using the NMR-L1 reporter revealed that NMR-L1 was active retrotransposon, but its activity was lower than that of human and mouse L1s. Despite lower retrotrasposition activity, NMR-L1 was still capable of inducing cell senescence, a tumor-protective system. NMR-L1 required the 3′ untranslated region (UTR) for retrotransposition, suggesting that NMR-L1 is a stringent-type of L1. We also confirmed the 5′ UTR promoter activity of NMR-L1. Finally, we identified the G-quadruplex structure of the 3′ UTR, which modulated the retrotransposition activity of NMR-L1. Taken together, the data indicate that NMR-L1 retrotranspose less efficiently, which may contribute to the cancer resistance of NMRs.

## Introduction

Naked mole-rats (NMRs; *Heterocephalus glaber*) are mouse-sized and the longest-living rodents discovered^1^. A unique feature of NMRs that contributes to their long lifespan is their pronounced cancer resistance, as there are only a few reports of spontaneous neoplasia in NMRs^1–4^. Several studies have sought to reveal the underlying mechanisms of NMR cancer resistance. For example, NMR cells transformed by oncogenic genes rapidly enter crisis or permanent cell cycle arrest, which may contribute to cancer resistance^5^. Activation of the tumor suppressor *ARF* and the disruption of oncogenic *ERAS* can also exhibit tumor resistance in NMR-induced pluripotent stem cells^6^. Another feature of NMRs that may be related to cancer resistance is fewer transposon-derived sequences than found in other mammals (25% in the NMR, 41% in the human, 38% in the mouse and 36% in the rat genomes)^7^. Transposons, such as endogenous retroviruses (ERVs) and long interspersed elements (LINEs), can autonomously amplify themselves in the genome^8^, which may produce oncogenic mutations and/or induce genomic instability, thereby promoting cancer development^9–13^. Thus, fewer transposon-derived sequences may indicate lower transposition activity of the NMR transposons, which may contribute to NMR cancer resistance.

LINE-1 (L1) is a transposon that is abundant in many eukaryotic genomes^8^. L1s constitute approximately 17.5% and 18.2% of the human and mouse genomes, respectively, while they constitute only 13.8% of the NMR genome^7^. This disparity raises the possibility that L1s in the NMR genome may amplify themselves less efficiently than those in the human and mouse genomes. L1s contain a 5′ untranslated region (UTR), two open reading frames (ORFs) that encode two proteins, ORF1p and ORF2p, and a 3′ UTR with a polyadenylation signal. ORF1p is an RNA-binding protein with nucleic acid chaperone activity that is required for L1 retrotransposition. ORF2p is a protein required for endonuclease (EN) and reverse transcriptase (RT) activity for a “copy-and-paste” retrotransposition of L1s to new genomic loci^14,15^. L1 retrotransposition occurs by target-site primed reverse transcription (TPRT), during which ORF2p makes a nick in genomic DNA and synthesizes L1 cDNA using the 3′ hydroxyl group at the nick^16^. The active retrotransposition of L1 is considered a major source of endogenous mutagenesis that can promote cancer development^9–13^. Consistently, we have found that Kaposi’s sarcoma-associated herpesvirus, an oncogenic virus, stimulates L1 retrotransposition, thereby enhancing transformation^17^. Together with the extraordinary resistance of NMR to cancer and the fewer transposon-derived sequences in the NMR genome, as described above, we reasoned that the retrotransposition of L1s in the NMR genome is less efficient than those in other genomes, which might contribute to cancer resistance. In this study, we cloned an active L1 from the NMR genome and characterized it to evaluate our hypothesis.

## Results

### Identification of an L1 sequence from the NMR genome

**Figure 1:**
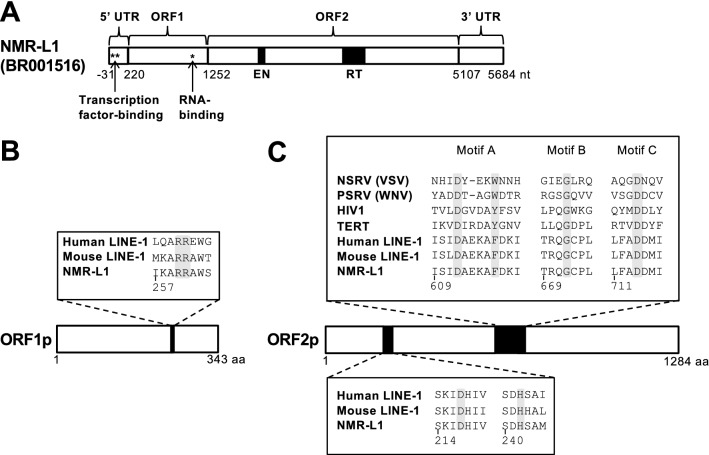
Identification of NMR-L1 from the naked mole-rat (NMR) genome. (**A**) Schematic representation of the NMR-L1 full sequence. Asterisks and black boxes are important regions for the indicated function. EN, endonuclease activity; RT, reverse transcriptase activity. (**B**) Sequence alignment of amino acids important for L1 RNA-binding in the NMR-L1-, mouse L1_spa_-encoded, and human L1_RP_-encoded ORF1p. (**C**) Sequence alignment of amino acids important for reverse transcriptase and endonuclease activities in the NMR-L1-, mouse L1_spa_-encoded, and human L1_RP_-encoded ORF2p together with other RNA-dependent polymerases. NSRV, negative-strand RNA virus; VSV, vesicular stomatitis virus; PSRV, positive-strand RNA virus; WNV, West Nile virus; HIV1, human immunodeficiency virus 1; and TERT, telomerase reverse transcriptase.

To identify an intact L1 sequence, which contained both potential ORF1p and ORF2p sequences, from the NMR genome, we searched the NMR genome database for nucleotide sequences with similarity to ORF2p of human L1_RP_ (accession no.: AAD39215) and mouse L1_spa_ (accession no.: AAC53542) using tblastn. The search using human L1_RP_ led to the identification of many potential ORF2p sequences (~ 3 kB) with a potential ORF1p (~ 1 kB) in the upstream region, while the search using mouse L1_spa_ found only short ORFs in the top 30 candidate sequences. Among these candidates identified by the search using human L1_RP_, we chose an intact L1 sequence and named it NMR-L1 (Fig. 1A). The accession number of the complete sequence of NMR-L1 identified in this study is BR001516. The nucleotide sequence identity of NMR-L1 with human L1_RP_ (accession no.: AF148856) was 63%. A comparison of the amino acid sequences of each gene showed 33% sequence identity and 79% similarity in the ORF1 gene and 63% identity and 92% similarity in the ORF2 gene. Phylogenetic analysis of the nucleotides coding ORF1p and ORF2p showed that NMR-L1 and human L1 Hs constituted a clade that was different from that of mouse L1s (Fig. S1). We compared the amino acid residues of NMR-L1 to those important for retrotransposition of human L1_RP_. All the residues we compared were conserved in the NMR-L1 ORF1p and ORF2p (Fig. 1, B and C). These results suggest that NMR-L1 likely exhibits intact retrotransposition activity.

### Characterization of NMR-L1 retrotransposition activity

**Figure 2:**
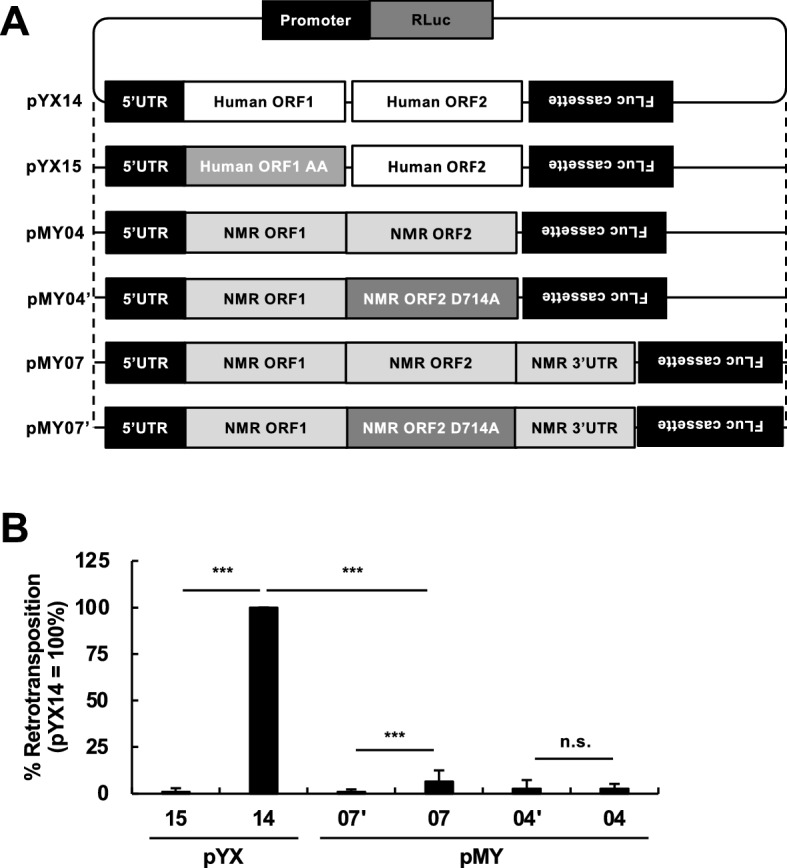
Characterization of the retrotransposition activity of NMR-L1. (**A**) Schematic view of the human L1_RP_ and NMR-L1 reporter plasmids. (**B**) The L1 retrotransposition activity of NMR-L1 with and without the 3′ UTR. 293T cells were transfected with the indicated L1 reporter plasmids. Luciferase activity level in the cells was evaluated at 4 days after transfection. The human L1 reporter plasmid (pYX14) was used as a positive control. Values are expressed as the means + S.E. of seven independent experiments. ***, *P* < 0.005.

To evaluate the retrotransposition activity of NMR-L1, we constructed NMR-L1 retrotransposition reporter plasmids (Fig. 2A). According to a previously established dual luciferase-based L1 retrotransposition assay^18^, we inserted the sequences of NMR-L1 (from ORF1 to the 3′ UTR, pMY07 and pMY07′; from ORF1 to ORF2, pMY04 and pMY04′) downstream of the 5′ UTR of L1_RP_ (Fig. 2A). We also inserted a *Firefly luciferase* (FLuc) reporter cassette in the antisense orientation, in which the FLuc gene was interrupted by an antisense intron, downstream of the 3′ UTR (Fig. 2A, pMY07 and pMY07′) or ORF2 (Fig. 2A, pMY04 and pMY04′). Thus, the FLuc activity was expected to be detected only after the donor L1 had undergone one round of retrotransposition. The *Renilla luciferase* (RLuc) gene in the plasmid was used to normalize the transfection efficiency. We also constructed FLuc-tagged NMR-L1 reporters with an D714A mutation in ORF2p (Fig. 2A, pMY07′ and pMY04′), which was expected to be defective in reverse transcription since an Asp in position 714 of the NMR-L1 ORF2p, which corresponds to an Asp in position 702 of the human L1 ORF2p, a critical residue for RT activity^19^, was substituted with an Ala residue (Fig. 1C). We used pYX14 as the reference human L1 reporter and pYX15 as the retrotransposition-defective L1 reporter (Fig. 2A)^18^. We then evaluated L1 retrotransposition using these constructed plasmids (Fig. 2B). Although the retrotransposition activity of NMR-L1 was markedly lower than that of a human L1, we detected significant retrotransposition activity by comparing the intact NMR-L1 (pMY07) with the NMR-L1-D714A mutant (pMY07′) (Fig. 2B). To evaluate the relevance in other cell lines, we investigated the retrotransposition activity of NMR-L1 in NMR SV40ER (an NMR fibroblast cell line; Fig. S2), 3T3 (a mouse fibroblast cell line), and OL (a human oligodendroglioma cell line) cells. In NMR SV40ER and 3T3 cells, we could not detect substantial retrotransposition activity of both human and NMR L1s (data not shown). On the other hand, in OL cells, we detected the retrotransposition activity of a human L1 but not NMR-L1 (Fig. S3), confirming that the retrotransposition activity of NMR-L1 is lower than that of a human L1. For comparison, we also constructed an FLuc-tagged reporter of mouse L1 (ORFeus-Mm)^20^, pMY10, and its defective mutant (ORFeus-Mm-D212G/D709Y)^20^, pMY11 (Fig. S4A). Mouse L1 showed more robust retrotransposition activity than a human L1 (Fig. S4B). These results suggest that retrotransposition activity of NMR-L1 is still low even if compared to a mouse L1. Then, to evaluate the contribution of EN activity to NMR-L1 retrotransposition, we constructed another reporter with an D217A mutation in ORF2p, which was expected to be defective in EN activity since an Asp in position 217 of the NMR-L1 ORF2p, which corresponds to an Asp in position 205 of the human L1 ORF2p^21^, a critical residue for EN activity, was substituted with an Ala residue (Fig. S5A). Using this reporter, we found that NMR-L1 defective for the EN activity showed lower retrotransposition activity than the wild-type (Fig. S5B), suggesting a critical role of EN activity in NMR-L1 retrotransposition. Furthermore, to test whether the cognate 3′ UTR was required for retrotransposition activity, we conducted an L1 retrotransposition assay using a FLuc-tagged NMR-L1 reporter lacking the cognate 3′ UTR (Fig. 2A, pMY04 and pMY04′). The FLuc activity levels of pMY04 and pMY04′ were comparable, suggesting that the NMR-L1 lacking the cognate 3′ UTR is retrotransposition incompetent (Fig. 2B). Taken together, our data show that we successfully cloned an active L1 sequence in the NMR genome, which had lower retrotransposition activity than human and mouse L1s and required the cognate 3′ UTR for retrotransposition.

### Characterization of the 5′ UTR of NMR-L1

**Figure 3:**
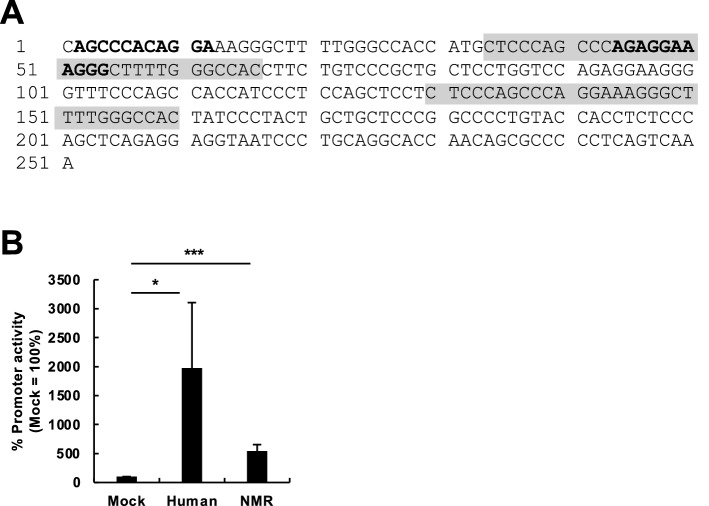
Characterization of the promoter activity of the 5′ UTR of NMR-L1. (**A**) Repeat sequences and transcription factor-binding sites in the 5′ UTR of NMR-L1. The gray boxes represent the repeated sequences with potential Runx1- and Erg-binding sites in bold. (**B**) The promoter activity of the 5′ UTR of NMR-L1. 293T cells were transfected with pGLuc-5′-UTR or pGLuc-NMR-L1-5′-UTR together with pCMV-CLuc. Luciferase activity level in the cells was evaluated at 3 days after transfection. pGLuc-5′-UTR, which contained the human L1 5′ UTR, was used as a positive control. Values are expressed as the means + S.E. of three independent experiments. *, *P* < 0.05; ***, *P* < 0.005.

The 5′ UTRs of L1s are promoters of L1 mRNA transcription^9^. Using JASPAR 2018^22^, we searched putative transcription factor-binding sites in the 5′ UTR of NMR-L1 and identified several transcriptional factors that may bind to the 5′ UTR, including Atoh1, Erg, Pitx1, NFkB1, and RUNX1 (Fig. 3A). We then subcloned the cognate 5′ UTR of the NMR-L1 into a pGLuc-Basic plasmid and measured the NMR-L1 5′ UTR promoter activity. Although the activity seemed lower than that of a human L1, we detected substantial promoter activity of the NMR-L1 5′ UTR (Fig. 3B). These results suggest that the NMR-L1 5′ UTR contains sequences with promoter activity.

### Characterization of the 3′ UTR of NMR-L1

**Figure 4:**
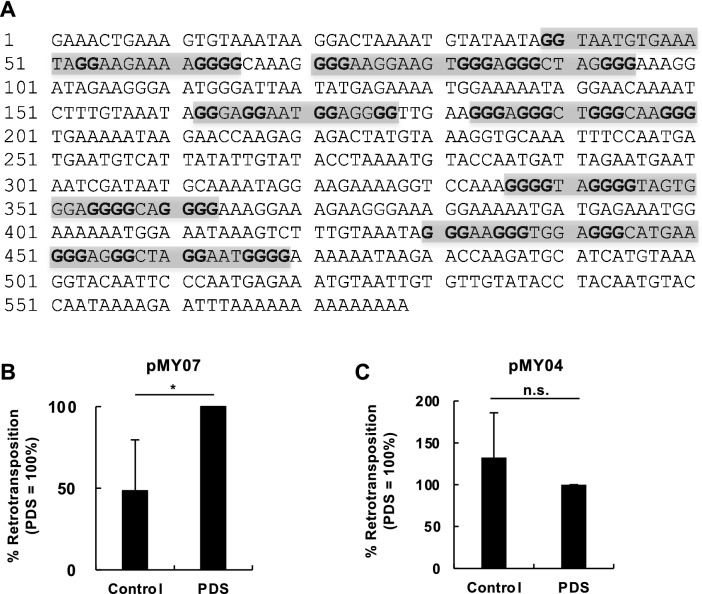
Enhancement of NMR-L1 retrotransposition by PDS. (**A**) Putative G-quadruplex (G4) sequences in the 3′ UTR of NMR-L1. The gray boxes indicate G4 sequences with Gs forming G4 structures in bold. (**B**, **C**) The L1 retrotransposition activity of NMR-L1 with (**B**) or without (**C**) the 3′ UTR in the presence of 200 nM PDS. 293T cells were transfected with the indicated L1 reporter plasmids. At 2 days after transfection, PDS was added to the transfected cells at a final concentration of 200 nM. Luciferase activity level in the cells was evaluated at 4 days after transfection. Values are expressed as the means + S.E. of seven independent experiments. *, *P* < 0.05.

Primate L1s contain guanine-rich sequences with the ability to fold into G-quadruplex (G4) structures in the 3′ UTR^23^. Because NMR-L1 is phylogenetically located near a human L1 (Fig. S1), we reasoned that NMR-L1 may contain guanine-rich sequences that can fold into G4 structures in the 3′ UTR, similar to human L1s. Consistent with our assumption, we found several guanine-rich sequences capable of forming G4 structures in the 3′ UTR of NMR-L1 (Fig. 4A). Stabilization of the G4 structures of human L1s reportedly stimulates L1 retrotransposition^23^. We therefore evaluated the effect of pyridostatin trifluoroacetate (PDS), a stabilizer of G4 structures, on NMR-L1 retrotransposition. NMR-L1 retrotransposition was enhanced by PDS treatment when NMR-L1 with the cognate 3′ UTR (pMY07) was used for the assay (Fig. 4B). On the other hand, the retrotransposition was unaffected by PDS when NMR-L1 without the 3′ UTR (pMY04) was used (Fig. 4C). These results suggest that the 3′ UTR of NMR-L1 contains G4 structures that modulate its retrotransposition activity.

### Cell senescence induction by NMR-L1 retrotransposition

**Figure 5:**
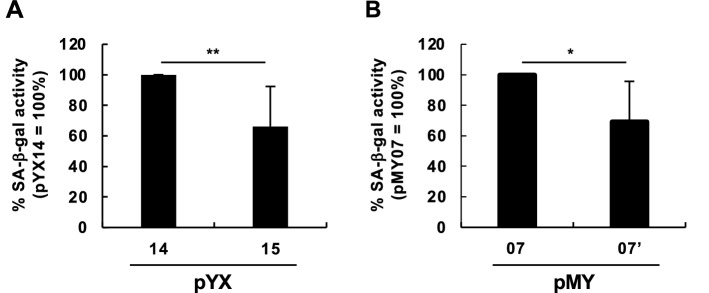
Cell senescence induced by NMR-L1 retrotransposition. (**A**, **B**) Cell senescence-associated $\upbeta$-galactosidase (SA-$\upbeta$-gal) activity induced by retrotransposition of a human L1 (**A**) and NMR-L1 (**B**). 293T cells were transfected with the indicated L1 reporter plasmids. SA-$\upbeta$-gal activity was evaluated at 3 days after transfection. Values are expressed as the mean + S.E. of four independent experiments. *, *P* < 0.05.

Cell senescence is one of tumor-protective mechanisms and also a hallmark of aging^24^. Although L1 retrotransposition drives oncogenic insertions, it can also induce cell senescence at least through stimulation of IFN responses^25^. To evaluate whether NMR-L1 induces cell senescence, we measured senescence-associated $\upbeta$-galactosidase (SA-$\upbeta$-gal) activity. As reported previously, we detected L1 retrotransposition-dependent cell senescence induction by a human L1 (Fig. 5A). Although NMR-L1 exhibited lower retrotransposition activity, it still induced cell senescence in a retrotransposition-dependent manner (Fig. 5B). These results suggest that NMR-L1 is less oncogenic because it is less competent to drive oncogenic insertions and does not affect the cell senescence-based tumor-protection.

## Discussion

NMRs are believed to have secrets of cancer resistance because they rarely develop cancer^1^. Previous studies have proposed several possible reasons for this resistance, including a highly efficient surveillance system of cell cycles and resistance to multiple stressors^5,26^. In addition, we reasoned that such secrets likely include low retrotransposition activity since the NMR genome contains fewer retrotransposons than other mammalian genomes^7^. L1 is a retrotransposon that is considered to be an endogenous mutagen, which can cause oncogenic mutations and contribute to cancer development^9–13^. In this study, we, for the first time, cloned an intact L1 from the NMR genome and determined its retrotransposition activity. The amino acids important for L1 retrotransposition in a human L1 appeared to be conserved in NMR-L1 (Fig. 1), suggesting that NMR-L1 can undergo retrotransposition in a manner similar to human L1s, i.e., possibly by TPRT^16^. The retrotransposition activity of NMR-L1 was indeed detectable but only at the minimum level compared to that of a human L1 (Fig. 2). These results are consistent with our hypothesis that L1s in the NMR genome undergoes retrotransposition less efficiently, which may contribute to the cancer resistance of NMRs, although we cannot exclude the possibility that other L1s in the NMR genome may exhibit strong retrotransposition activity. Alternatively, L1s in the NMR genome may have evolved to function within the confines of the biology unique to NMRs. Although we sought to evaluate L1 rtrotranspositon activity in mouse and NMR cells, we could not detect substantial retrotransposition activity of both a human L1 and NMR-L1 in mouse 3T3 and NMR SV40ER cells (data not shown). This might be due to strong anti-L1 activity in NMR cells. In any case, our results at least indicate that NMR-L1 does not exhibit robust retrotransposition even in NMR cells.

The 3′ UTR of human and primate L1s is dispensable for retrotransposition; however, we found that the 3′ UTR was required for NMR-L1 retrotransposition (Fig. 4), suggesting that NMR-L1 is a stringent-type L1 that strictly recognizes its own 3′ UTR for retrotransposition. The RNAfold web server (http://rna.tbi.univie.ac.at/cgi-bin/RNAWebSuite/RNAfold.cgi) predicted the secondary structure of the 3′ UTR of NMR-L1 (Fig. S6). Since the 3′ UTRs of primate L1s are devoid of canonical secondary structures^23^, the 3′ UTR secondary structures likely play a role in NMR-L1 retrotransposition, and thus, the 3′ UTR was required for it. Although the 3′ UTRs of primate L1s are thought to lack a secondary structure, they reportedly contain conserved guanine-rich sequences that can form G4 structures^23^. Similarly, we found that the 3′ UTR of NMR-L1 contained G4 structures (Fig. 4A). G4 structures are noncanonical secondary structures formed by guanine-rich nucleic acids and stabilized by stacks of guanine tetrads held together by Hoogsteen base pairing^27^. Stabilization of the G4 motif in a human L1 stimulates its retrotransposition^23^. Consistent with this, the stabilization of the G4 motif in the 3′ UTR of NMR-L1 also stimulated retrotransposition (Fig. 4, B and C). Together with the enrichment of G4 motifs in human L1s, nonautonomous Alu and SVA elements, and retroviral long terminal repeats, our finding supports the idea that the G4 motifs may be drivers of gene copy-number variation and horizontal gene transfer^23^.

The 5′ UTR of NMR-L1 contained ~ 30-bp repeat sequences (Fig. 3A). Mouse L1 Tf contains a tandem repeat of monomer unit (~ 200 bp) in the 5′ UTR, and an eel LINE, UnaL2, has a series of 39-bp repeats^28,29^. These repeat sequences are important for promoter activity^29^, suggesting that the 5′ UTR of NMR-L1 may also have activity. Indeed, the 5′ UTR promoter activity of NMR-L1 was demonstrated by NMR-L1 promoter assay (Fig. 3). We found the binding sites of 12-O-tetra-decanoylphorbol-13-acetate (TPA)-related transcription factors, RUNX1 and Erg^30^, in the 5′ UTR of NMR-L1 (Fig. 3A), suggesting that NMR-L1 was actively transcribed possibly through the binding of these transcriptional factors. Since TPA is a potent activator of protein kinase C, which regulates various biological processes, such as cell proliferation and vascular development, NMR-L1 expression might be upregulated during developmental processes, such as angiogenesis^31^.

Although enhanced L1 retrotranspositon is considered to promote oncogenic transformation by insertional mutations, it is also reported that L1 retrotransposition induces cell senescence, a tumor-protective system and also a hallmark of aging. In this study, we demonstrated that NMR-L1 can induce cell senescence, similarly to a human L1 despite its low retrotransposition activity. This suggests that NMR-L1 is less oncogenic in total than a human L1 because it has lower retrotransposition activity but still can stimulate a tumor-protective system. Since we could not activate L1 retrotransposition in NMR cells, it is unclear whether enhanced L1 retrotransposition indeed promotes transformation in NMR cells. However, as we have already demonstrated that enhanced L1 retrotranspositon induces transformation in human cells^17^, it is reasonable to speculate that lower retrotranspositon of NMR-L1 contributes to low incidence of cancer in NMRs.

In conclusion, we successfully developed an L1 retrotransposition reporter system for NMR-L1. Using this system, we demonstrated that NMR-L1 exhibited lower retrotransposition activity than other L1s, which might be a reason of the cancer resistance of NMRs. If this is the case, then our findings suggest the importance of the regulation of L1 retrotransposition for preventing cancer development, and screening of chemicals that suppress L1 retrotransposition, such as capsaicin^32^, may facilitate the development of ways of cancer prevention.

## Materials and methods

### Cells

293T cells (a human embryonic kidney cell line from ATCC), OL cells (a human oligodendroglioma cell line^33^), and 3T3 cells (a mouse fibroblast cell line from ATCC) were cultured in Dulbecco’s modified Eagle’s medium (DMEM) supplemented with 5%, 5%, and 10% fetal bovine serum (FBS), respectively. NMR SV40ER cells (an NMR fibroblast cell line; also see below) were cultured in DMEM supplemented with 15% FBS, non-essential amino acids (Gibco) and L-glutamine (Nakalai Tasque, Kyoto, Japan).

### Establishment of NMR SV40ER cells

Primary NMR fibroblasts were isolated from back skin of 1-year-old adult NMR. Fibroblasts were cultured at 32 °C in a humidified atmosphere containing 5% CO_2_ and 5% O_2_. NMR fibroblasts expressing simian virus 40 early region (SV40ER) were generated by lentiviral infection with the pCSII-EF-SV40ER-TK-hyg vector [The backbone vector (pCSII-EF-RfA-TK-Hyg) was kindly provided by Dr. Hayato Naka-Kaneda (Shiga University of Medical Science, Japan)] and selection of hygromycin-resistant cells for 4 days. Subsequently, cloning by limiting dilution was performed, and stable clones (NMR SV40ER cells) were established. Animal experiments were done in accordance with protocols approved by the Institutional Animal Care and Use Committee in Kumamoto University (A2020-042) and in compliance with the ARRIVE guidelines.

### Plasmids

pYX014 and pYX015, reporter plasmids for a human L1 retrotransposition, were kindly provided by Dr. Wenfeng An (South Dakota State University, USA)^18^. pYX015 is a mutant L1 construct defective for L1 retrotransposition^18^. The reporter plasmids for a mouse L1 retrotransposition were prepared by substituting a human L1 sequence of pYX14 with ORFeus-Mm or ORFeus-Mm-D212G/D709Y, which was kindly provided by Dr. Jef D Boeke (NYU Langone Health, USA)^20^. The reporter plasmids for NMR-L1 retrotransposition were prepared by substituting human L1 sequences with partially codon-optimized NMR-L1 ORF1 and ORF2 sequences with or without the 3′ UTR of NMR-L1. The reporter plasmids with a putative retrotransposition-defective mutation were prepared by site-directed mutagenesis using PrimeSTAR Max DNA polymerase (TaKaRa, Shiga, Japan). Summary descriptions of the constructed plasmids are shown in Figs. 2A, S4A, and S5A. The reporter plasmid for NMR-L1 promoter activity, pGLuc-NMR-5′-UTR, was generated by subcloning the 5′ UTR of NMR-L1 into a pGLuc-Basic plasmid (New England Biolabs, Ipswich, MA, USA). The reporter plasmid for human L1 promoter activity, pGLuc-5′-UTR, was generated previously^32^.

### L1 retrotransposition assay

293T cells were transfected with retrotransposition reporter plasmids using Lipofectamine 2000 or 3000 reagent (Invitrogen). At 4 days after transfection, *Firefly* and *Renilla* luciferase activity levels were measured using a Dual-Luciferase Reporter Assay System (Promega, Fitchburg, MA, USA) according to the manufacturer’s instructions in a single-well luminometer (Berthold, Lumat LB 9507, Bad Wildbad, Germany). The *Firefly* luciferase activity level was normalized to the corresponding *Renilla* luciferase activity level. Then, the activity was further normalized to that of pYX14. PDS (Selleck, Houston, TX, USA) was added to the cells at 200 nM at 2 days after transfection.

### L1 promoter assay

293T cells were cotransfected with pGLuc-5′-UTR (human) or pGLuc-NMR-L1-5′-UTR, together with pCMV-CLuc (New England Biolabs) using Lipofectamine 2000 reagent. At 3 days after transfection, the *Gaussia* and *Cypridina* luciferase activity levels were measured using *Gaussia* and *Cypridina* Luciferase Assay Kits (New England Biolabs) according to the manufacturer’s instructions. *Gaussia* luciferase activity level was normalized to the corresponding *Cypridina* luciferase activity level. Then, the activity was further normalized to that of pGLuc-Basic (Mock).

### Cell senescence assay

293T cells were transfected with pYX14, pYX15, pMY07, or pMY07′ using Lipofectamine 2000 reagent. At 3 days after transfection, SA-$\upbeta$-gal activity was measured using a 96-Well Cellular Senescence Assay Kit (Cell Biolabs, San Diego, CA) according to the manufacturer’s instructions. SA-$\upbeta$-gal activity level was normalized to the corresponding *Renilla* luciferase activity level. Then, the activity was further normalized to that of pYX14 or pMY07.

### Statistics

Statistical significance was assessed using a two-tailed Student’s *t*-test with a threshold of *P* < 0.05.

## Supplementary Information

Supplementary Information
