# Supplementary material for: Characterization of an active LINE-1 in the naked mole-rat genome
Source: Sci Rep. 2021 Mar 11;11:5725. doi: 10.1038/s41598-021-84962-8 (PMC7952902; doi:10.1038/s41598-021-84962-8)
Supplement: Supplementary file 1 — Supplementary Information [file 41598_2021_84962_MOESM1_ESM.pdf]

Supplementary Information

Characterization of an active LINE-1 in the naked mole-rat genome

Shunichi Yamaguchi, Shizuka Nohara, Yuki Nishikawa, Yusuke Suzuki, Yoshimi Kawamura, Kyoko Miura, Keizo Tomonaga, Keiji Ueda, and Tomoyuki Honda

Fig. S1-S6.

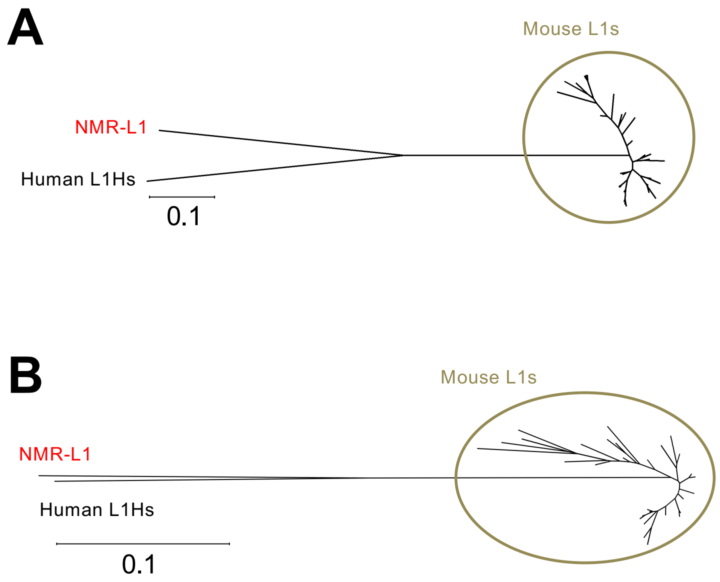

Figure S1. Phylogenetic analysis of L1 sequences. The phylogenetic analysis of L1 ORF1p (A) or ORF2p (B) sequences of L1s in the human, mouse, and NMR genomes was conducted using MEGA7 software. The sequences of mouse L1s were obtained from Repbase (https://www.girinst.org/repbase/).

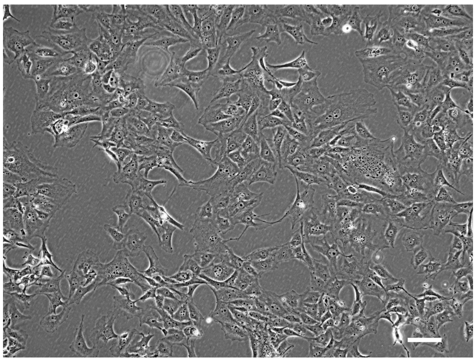

Figure S2. Establishment of NMR SV40ER cells. Representative image of NMR SV40ER cells. Bar, 100 μm.

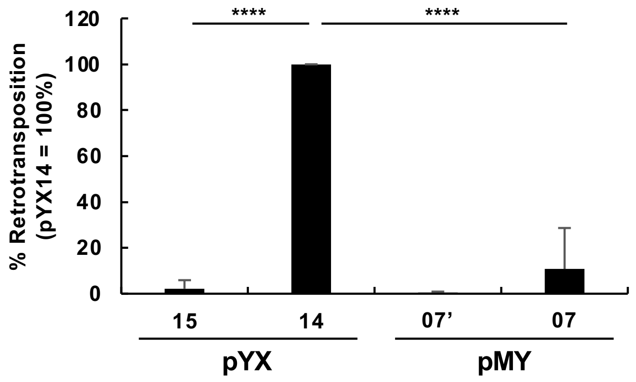

Figure S3. Retrotransposition activity of NMR-L1 in OL cells. OL cells were transfected with the indicated L1 reporter plasmids. Luciferase activity level in the cells was evaluated at 4 days after transfection. The human L1 reporter plasmid (pYX14) was used as a positive control. Values are expressed as the means + S.E. of three independent experiments. ****, $P < 0.001$.

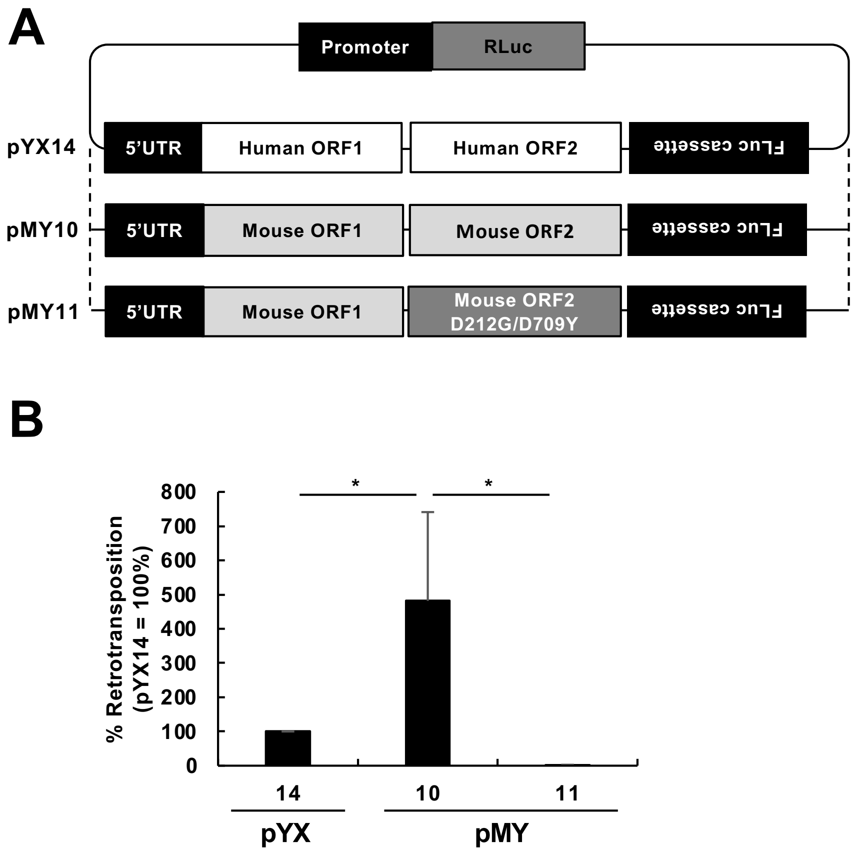

Figure S4. Characterization of the retrotransposition activity of a mouse L1. (A) Schematic view of the human $L1_{RP}$ and mouse L1 (ORFeus_Mm) reporter plasmids. (B) The L1 retrotransposition activity of a mouse L1. 293T cells were transfected with the indicated L1 reporter plasmids. Luciferase activity level in the cells was evaluated at 4 days after transfection. The human L1 reporter plasmid (pYX14) was used as a positive control. Values are expressed as the means + S.E. of four independent experiments. *, $P < 0.05$.

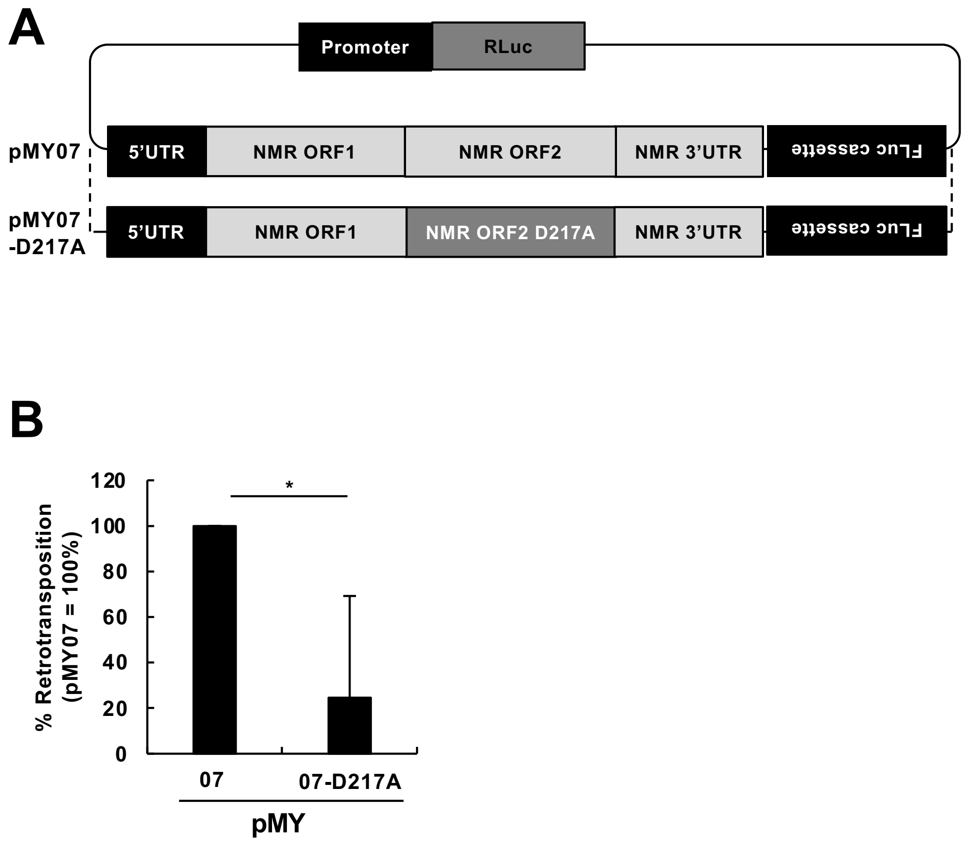

Figure S5. Retrotransposition activity of NMR-L1 with an ORF2 D217A mutation. (A) Schematic view of the NMR-L1 reporter plasmids with or without an ORF2 D217A mutation. (B) The L1 retrotransposition activity of NMR-L1 with an ORF2 D217A mutation. 293T cells were transfected with the indicated NMR-L1 reporter plasmid. Luciferase activity level in the cells was evaluated at 4 days after transfection. Values are expressed as the means + S.E. of four independent experiments. *, $P < 0.05$.

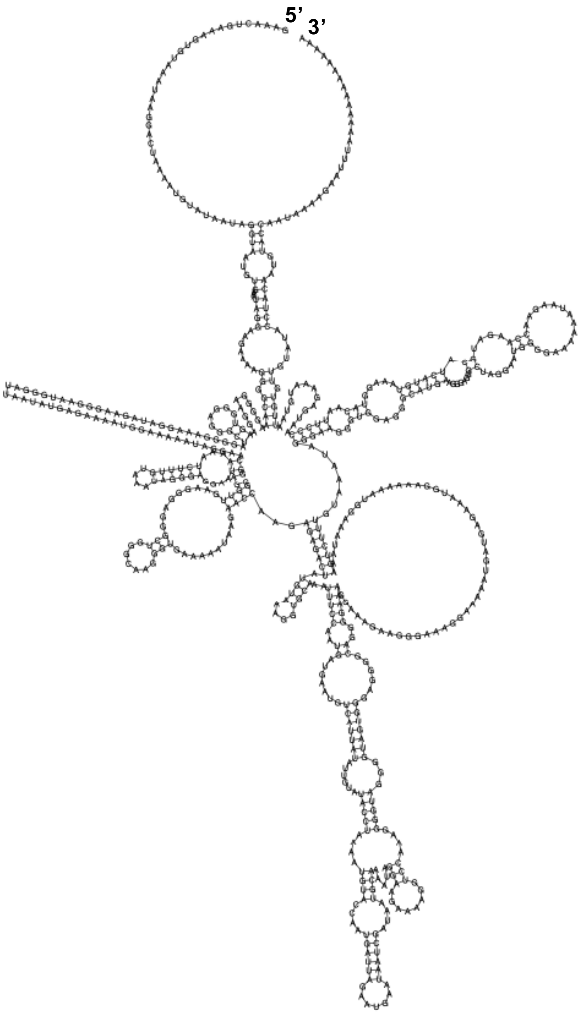

Figure S6. Predicted RNA secondary structure of the 3’ UTR of NMR-L1. Secondary structure of the 3’ UTR of NMR-L1 mRNA was predicted using RNAfold software (http://rna.tbi.univie.ac.at/cgi-bin/RNAWebSuite/RNAfold.cgi).
